# Healthcare Resource Consumption and Related Costs of People Living with HIV and Antiviral Treatment: A Retrospective Observational Study in Italy

**DOI:** 10.3390/diseases14030110

**Published:** 2026-03-18

**Authors:** Luca Degli Esposti, Stefania Mazzoni, Maria Cappuccilli, Franco Maggiolo, Sergio Lo Caputo, Silvia Nozza, Lucia Taramasso, Anna Marra, Mario Pittorru

**Affiliations:** 1 CliCon S.r.l., Società Benefit-Health, Economics and Outcomes Research, 40137 Bologna, Italy; stefania.mazzoni@clicon.it (S.M.); maria.cappuccilli@clicon.it (M.C.); 2 Independent Researcher, 05015 Fabro, Italy; franco31556@gmail.com; 3 Infectious Disease Unit, A.O.U. Policlinico di Foggia, University of Foggia, 71122 Foggia, Italy; sergio.locaputo@unifg.it; 4 Infectious Diseases Unit, Vita-Salute San Raffaele University, 20132 Milan, Italy; nozza.silvia@hsr.it; 5 Infectious Diseases Unit, IRCCS San Raffaele Scientific Institute, 20127 Milan, Italy; 6 Infectious Diseases Clinic, IRCCS Policlinico San Martino Hospital, 16132 Genova, Italy; taramasso.lucia@gmail.com; 7 Pharmaceutical Department, Azienda Ospedaliero-Universitaria di Ferrara, 44121 Ferrara, Italy; a.marra@ospfe.it; 8 Continuity Pharmacy, Department of Pharmacy, Tuscany Central Health Authority, 50122 Firenze, Italy; mario.pittorru@uslcentro.toscana.it

**Keywords:** antiretroviral therapies, bictegravir/emtricitabine/tenofovir alafenamide, medication adherence, people living with HIV, persistence in therapy, real-world evidence

## Abstract

Background/Objectives: Among the antiretroviral therapies (ARTs) recently introduced for people living with HIV (PLWH), the fixed-dose combination of bictegravir, emtricitabine and tenofovir alafenamide (B/F/TAF) became reimbursable in Italy in June 2019. Methods: This study evaluated drug utilization, healthcare resource consumption and direct costs among ART-naïve adults initiating B/F/TAF or other non-bictegravir-based regimens, identified from June 2019 to September 2022 within administrative databases of healthcare entities covering approximately nine million citizens. Baseline clinical characteristics at first ART prescription were compared across B/F/TAF-treated patients, those receiving other ART regimens, and non-HIV controls, while treatment outcomes during follow-up were evaluated among PLWH receiving B/F/TAF or other ARTs; healthcare consumption and costs were assessed after propensity score matching within the PLWH cohorts only. Results: Overall, 374 individuals initiated B/F/TAF and 5576 other ARTs. Patients treated with B/F/TAF showed greater adherence and persistence, with multivariate analyses confirming a lower risk of discontinuation or switching (HR = 0.66, 95% CI 0.57–0.76, *p* < 0.001) and a higher likelihood of adherence (HR = 2.40, 95% CI 1.58–3.64, *p* < 0.001). After matching, the B/F/TAF group exhibited lower 12-month consumption of non-HIV medications, fewer non-HIV hospitalizations, and reduced total healthcare costs, particularly for non-HIV drug prescriptions compared to other ART users. Conclusions: Overall, B/F/TAF was associated with better treatment continuity and meaningful cost savings.

## 1. Introduction

Since the first reported cases of HIV infection in 1981 [1], extraordinary improvements have been accomplished in care, quality of life and life expectancy of people living with HIV (PLWH) [2,3,4,5].

Undeniably, the advent of highly effective antiretroviral therapies (ARTs) in 1996 has been a turning point in the history of HIV infection [1]. ARTs not only provided a nearly normal life expectancy to PLWH, but also minimized the risk of virus transmission to an uninfected sexual partner [6,7]. Nevertheless, these benefits can be successfully achieved in those people who receive early diagnosis, timely treatment and who are compliant with their therapy [8,9,10].

As in other chronic clinical diseases, PLWH may experience multiple symptoms and are often burdened by concomitant conditions that require complex therapies with multiple drugs in addition to ARTs [3]. Unfortunately, polypharmacy regimens result in higher likelihood of adverse events, drug–drug interactions (DDIs) and imply more challenging self-management from the patient’s perspective [11,12,13]. Evidence has shown how multiple comorbidities with complex therapeutic schedules represent a barrier to treatment adherence and increase the risk of discontinuation [14,15].

With a view of optimizing therapy and alleviating pill burden, ART combinations [16] have been made available as single-tablet regimens (STRs) since 2006 [16,17,18], resulting in better adherence and lower discontinuation rates over multi-tablet regimens (MTRs) [19,20] and ultimately providing substantial clinical benefits for PLWH in terms of higher life expectancy and reduced hospitalizations [21,22]. Thus, optimizing the therapy through STRs has emerged as a valuable strategy not only to improve drug utilization and PLWH’s outcomes, but also to alleviate cost burden from the perspective of the National Health System (NHS). We have recently reported that the use of a TAF-based therapy in PLWH was associated with progressively decreased healthcare resource consumption and costs—other than HIV-related drugs themselves—highlighting a virtuous circle between the availability of novel therapeutic options for PLWH, better drug utilization, and cost savings due to reduced HIV-related hospitalizations [22].

Among the newly available STRs, the combination of bictegravir, a second-generation integrase inhibitor (INI), with emtricitabine and tenofovir alafenamide (B/F/TAF) has been approved for reimbursement in Italy since June 2019 for the treatment of adults with HIV infection without present or past evidence of viral resistance to INIs, emtricitabine or tenofovir [23].

The growing spread of B/F/TAF in the normal clinical practice might generate valuable evidence by real-life data, with the dual advantage of including people poorly represented in clinical trials and providing insights for the potential cost savings for the Italian NHS. On the other hand, given its relatively recent approval, evidence of the clinical and economic rebounds of B/F/TAF advent is still scanty. In this framework, an intriguing question is to assess whether a person adherent and persistent to B/F/TAF might reach comparable healthcare resource utilization and related costs (drug costs excluded) as the general HIV-negative population.

This analysis was aimed at describing the characteristics of PLWH treated with B/F/TAF or other ARTs in Italy to evaluate adherence and persistence on B/F/TAF compared to other ARTs and the healthcare resource consumptions and costs.

## 2. Materials and Methods

### 2.1. Data Source

A retrospective observational analysis was conducted using the administrative flows of a pool of Italian Local Health Units (LHUs), covering around 9 million health-assisted subjects with data available from January 2010 to September 2022 (at least). The following databases were used: (1) demographic database to collect data on age, sex and death (if applicable); (2) pharmaceutical database, to collect information related to drug prescriptions, including Anatomical-Therapeutic Chemical (ATC) code, number of packages, number of units per package, cost and prescription date; (3) hospitalization database, for all hospitalization data with discharge diagnosis codes classified according to the International Classification of Diseases, Ninth Revision, Clinical Modification (ICD-9-CM), Diagnosis-Related Group (DRG) and DRG-related charge; (4) outpatient specialist services database, to collect data on provision, type, and description of diagnostic tests and specialist visits.

The dataset used consists solely of anonymized data. All the results of the analyses were produced and presented as aggregated summaries. Approval has been obtained from the ethics committees of the participating healthcare entities.

### 2.2. Study Design and Population

The analysis included all adult (≥18 years old) people treated with ARTs from June 2019 up to the latest data available at the time of data extraction (to date, September 2022).

ART prescriptions were identified by specific ATC codes for protease inhibitors (ATC code J05AE), nucleoside and nucleotide reverse-transcriptase inhibitors (ATC code J05AF), non-nucleoside reverse-transcriptase inhibitors (ATC code J05AG), integrase inhibitors (ATC code J05AJ), combinations of antivirals for treatment of HIV infections (ATC code J05AR), and other antivirals (ATC codes J05AX07, J05AX09, J05AX12, J05AX23, J05AX29). B/F/TAF prescriptions were identified by the ATC code J05AR20. The time of the first ART prescription was considered the date of inclusion (index-date).

People with only one ART prescription, or with gaps between two consecutive ART prescriptions above 12 months, or with less than one year of data available before and after the index-date were excluded. A control group of HIV-negative adult subjects, identified by the absence of ART prescriptions, matched for sex, age, and calendar year of index date was gathered to provide contextual comparison of demographic and clinical characteristics between PLWH and the general population.

### 2.3. Study Variables

The demographic and clinical characteristics of the study participants were investigated at index-date or in the period before inclusion, as appropriate. Demographic variables, collected at index-date, were age (years) and sex distribution expressed as the percentage of male subjects.

The general clinical status of the included subjects was investigated in the period prior to inclusion using a modified version of the Charlson comorbidity index (CCI) not accounting for HIV [24]. Concomitant conditions were searched for in the administrative databases by means of specific hospitalization codes and drug prescriptions as diagnosis proxy.

Then, comorbidities most frequently reported for PLWH [25,26] were extrapolated through hospitalization discharge codes (ICD-9-CM) or active exemption codes searched for during all the available periods before the index-date, or specific drug prescriptions searched for in the 12-month period before the index-date. Explicitly, the following conditions were considered: depression (at least one prescription for ATC code N06A); respiratory disease (at least one prescription for ATC code R03 or at least a hospitalization with ICD-9-CM code 460–519 or exemption codes 007, 057); renal failure (at least a hospitalization with ICD-9-CM code 584–586 or exemption code 023); alcohol/drugs abuse (at least a hospitalization with ICD-9-CM code 303–304 or active exemption code 014); osteoporosis (at least one prescription for ATC code M05BA, M05BB, M05BX, H05AA, H05BA, G03XC, or at least a hospitalization with ICD-9-CM code 733.0); cardiovascular disease (at least a hospitalization with ICD-9-CM code 410–414, 431, 430, 432–438, 440, 443); diabetes (at least one prescription for ATC code A10, at least a hospitalization with ICD-9-CM code 250 or exemption code 013); dyslipidemia (at least 2 prescriptions for ATC code C10); HBV/HCV (at least a hospitalization with ICD-9-CM code 070.2, 070.3, 070.7 or active exemption code 016); hypertension (at least 2 prescriptions for ATC code C02, C03, C07, C08, C09, at least a hospitalization with ICD-9-CM code 401–405 or exemption code 0031); cancer (at least a hospitalization with ICD-9-CM code 140–239 or exemption code 048).

PLWH were classified as ART-naïve if no previous ART prescription was found in the database during all available characterization periods (at least 12 months preceding inclusion), otherwise they were deemed as ART-experienced.

Drug utilization, healthcare resource consumption and the resulting direct costs were assessed during follow-up.

Adherence was estimated during the first 12 months of follow-up as proportion of days covered (PDC), namely the ratio between the number of days of medication supplied and days of therapy, multiplied by 100. For the definition of adherence two cut-offs were used, PDC ≥ 85% and PDC ≥ 95%, and all the related analyses were conducted in parallel with both cut-offs. The assessment of adherence was made on alive subjects without a switch or discontinuation of therapy.

Discontinuation, defined as the absence of index ART therapy for at least 3 consecutive months, was evaluated during all the available follow-up. Switch, defined as the change of index ART therapy, was also examined during the entire follow-up; changes from STR to an equivalent combination of MTR were not considered a switch.

Healthcare resources reimbursed by the Italian NHS were computed as the mean number per person during the first 12 months of follow-up in terms of prescriptions for non-HIV-related medications, non-HIV-related hospitalizations (i.e., excluding DRG 488, 489, 490), and outpatient services (specialist visits/diagnostic tests) on alive subjects without therapy switches.

The direct healthcare costs derived per person were also assessed during the first 12 months of follow-up among live non-switching study participants. Costs were calculated as overall expenses and for each of the aforementioned items.

### 2.4. Statistical Analysis

Continuous variables were described using mean ± standard deviation (SD) and, where appropriate, median and interquartile range (IQR), while categorical variables were described as numbers and percentages. Comparisons of categorical variables were performed using chi-square tests. Continuous variables were compared using Student’s *t*-test for mean values and the Mann–Whitney U test for median values in order to account for potentially skewed distributions.

According to Opinion 05/2014 on Anonymisation Techniques drafted by the European Commission Article 29 Working Party, analyses involving fewer than 3 patients were not reported as they could potentially allow re-identification of single individuals. Therefore, results referring directly or indirectly to ≤3 patients were reported as NI (not issuable).

Comparative analyses were conducted PLWH treated with other ARTs (other ART cohort), PLWH treated with B/F/TAF (B/F/TAF cohort), and non-HIV controls (for baseline variables). In order to minimize selection bias, the groups were balanced with propensity score matching (PSM), using 1:1:1 ratio, in relation to age, sex, CCI, concomitant conditions and year of inclusion. The standardized mean difference (SMD) between each variable was computed, and an SMD < 0.2 was considered to define that variable as comparable [27].

Kaplan–Meier curves with median and interquartile range (IQR) were applied to analyze discontinuation and switch. In order to control confounding factors, a Cox regression model was implemented for the potential predictors of discontinuation and switch. Two logistic regression models, adjusted for confounding factors, were run to identify potential predictors of increased or decreased adherence with both cut-offs, PDC ≥ 85% and PDC ≥ 95%.

Healthcare resource consumption and direct costs were limited to non-HIV-related medications, hospitalizations, and outpatient services, explicitly excluding antiretroviral therapy costs. All cost and utilization comparisons between ART regimens were conducted after PSM to balance demographic and clinical comorbidities outliers, namely those values deviating more than three times the standard deviation from the mean, and were excluded from the cost analysis.

A *p* value < 0.05 was considered statistically significant, and the analyses were conducted using Stata SE version 17.0 (StataCorp, College Station, TX, USA).

## 3. Results

### 3.1. Study Population

During the inclusion period, 374 people who started B/F/TAF treatment and 5576 who started other ARTs were identified. As shown in Table 1, naïve people starting B/F/TAF were younger (mean 46.8 ± 11.8 and 59.5 ± 15.7 years, respectively), were more frequently males (77.8% and 62.6%), had a lower CCI (0.2 ± 0.6 and 1.2 ± 1.7), and a less common occurrence of HBV/HCV infection, dyslipidemia, hypertension and cancer, compared to other ARTs.

### 3.2. Drug Utilization

**Figure 1:**
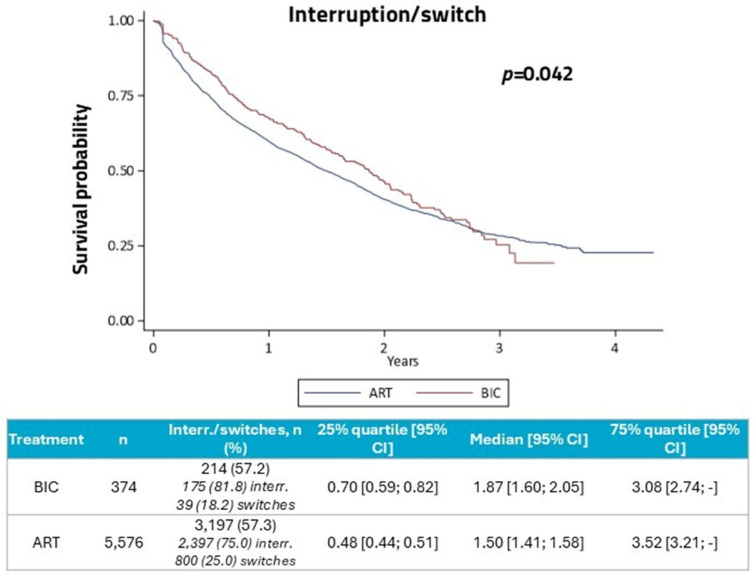
Kaplan–Meier survival analysis for therapy interruptions/switches in B/F/TAF group and other ART group. Abbreviations: ART, antiretroviral therapy; B/F/TAF, bictegravir/emtricitabine/tenofovir; CI, confidence interval.

The rate of interruptions/switches was similar between B/F/TAF and other ART groups (57.2% and 57.3%, respectively. Kaplan–Meier survival analysis (Figure 1) showed that the median time on therapy was 1.87 years for the B/F/TAF group and 1.50 years for the other ART group (*p* = 0.042).

The Cox regression model adjusted for confounding factors revealed that B/F/TAF-treated PLWH had the risk of discontinuation or switch decreased by 34% compared to other ARTs. Older age, HBV/HCV and cancer were predictors of lower risk of discontinuation/switch, while renal failure was associated with a higher risk (Table 2).

**Figure 2:**
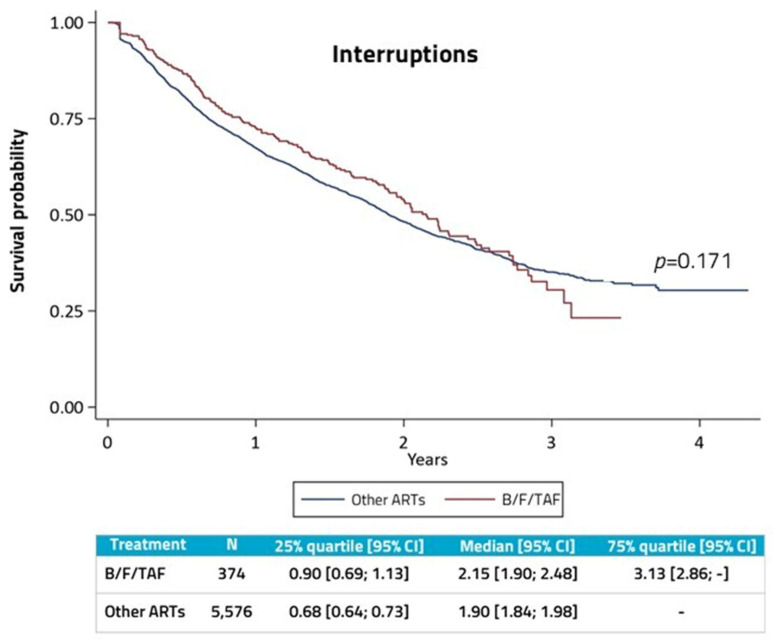
Kaplan–Meier survival analysis for therapy interruption in B/F/TAF group and other ART group. Abbreviations: ART, antiretroviral therapy; B/F/TAF, bictegravir/emtricitabine/tenofovir; CI, confidence interval.

Interruptions and switches were then analyzed separately. Considering only interruptions, Kaplan–Meier survival analysis (Figure 2) showed that the median time on therapy was 2.15 years for the B/F/TAF group and 1.90 years for the other ART group (*p* = 0.171).

The Cox regression model adjusted for confounding factors revealed that B/F/TAF-treated PLWH had a risk of discontinuation decreased by 29% compared to those on other ARTs (*p* < 0.023). The variables significantly associated with lower risk of discontinuation were older age (45–59 years and 60–79 years, *p* < 0.001; age ≥ 80 years, *p* = 0.018), male sex (*p* = 0.024) and HBV/HCV infection (*p* = 0.001), while renal failure increased the risk of discontinuation (*p* = 0.002) (Table 3).

Regarding the analysis of switches, the Cox regression model adjusted for confounding factors revealed that the risk of B/F/TAF-treated patients switching to another therapy decreased by 70% compared to other ART PLWH (*p* < 0.001). The variables significantly associated with a lower risk of switch were age over 45 years (*p* < 0.001), CCI above 0 (CCI = 1, *p* = 0.012; CCI ≥ 2, *p* < 0.001), HBV/HCV infection (*p* < 0.001), and cancer (*p* < 0.001), while renal failure was associated with an increased risk of switch (*p* = 0.010) (Table 4).

**Figure 3:**
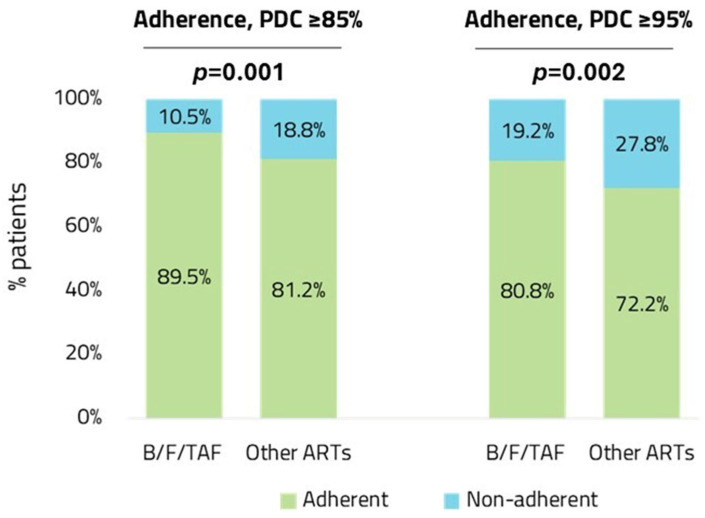
Relative proportions of adherent and non-adherent people in B/F/TAF vs. other ART groups, with PDC ≥ 85% and PDC ≥ 95% as cut-offs for adherence. Abbreviations: ART, antiretroviral therapy; B/F/TAF, bictegravir/emtricitabine/tenofovir; PDC, proportion of days covered.

The evaluation of adherence over 12 months of follow-up indicated that the B/F/TAF group had a higher percentage of adherent PLWH compared to the other ART group. Considering PDC ≥ 85% as the cut-off for adherence, the proportion of adherent subjects was 90% and 81% in B/F/TAF and other ART groups, respectively (*p* = 0.001); when using PDC ≥ 95% as the cut-off for adherence, the proportion of adherent subjects was 81% and 72% in B/F/TAF and other ART groups, respectively (*p* = 0.002) (Figure 3).

Logistic regression models adjusted for confounding factors for predictors of adherence showed that with both cut-offs (PDC ≥ 85% and PDC ≥ 95%), renal failure was associated with a lower probability of being adherent (*p* < 0.001), while HBV/HCV infection was predictive of higher adherence (*p* < 0.001) (Table 5).

PSM was then applied to balance the B/F/TAF group, the other ART group and a non-HIV-control population by age, sex, index-year and comorbidities. Thus, 321 subjects were identified for each cohort. Even though all the baseline variables were balanced across groups, a slight unbalance was found for male sex (SMD = 0.31) (Table 6).

### 3.3. Healthcare Resources Consumption and Direct Costs

Healthcare resources consumption in PSM-matched cohorts of PLWH during the first 12 months of follow-up is described in Table 7. The B/F/TAF group showed a lower number of prescriptions for non-HIV-related drugs compared to the other ART-treated group (6.0 ± 8.4 vs. 8.8 ± 12.2, *p* < 0.001) and required less hospital admissions for other causes, non-HIV-related (0.2 ± 0.5 vs. 0.3 ± 0.8, *p* = 0.050). Outpatient services were comparable between the two groups (*p* = 0.228).

Direct healthcare costs per person (outliers excluded) in PSM-balanced cohorts of PLWH during the first 12 months of follow-up are shown in Table 8. B/F/TAF vs. other ART users had lower total costs per person (€2078 ± 3292 vs. €3202 ± 5849, *p* = 0.003), and a lower cost of prescriptions for non-HIV-related drugs (€572 ± 1853 vs. €1553 ± 4225, *p* < 0.001).

## 4. Discussion

This analysis investigated the population of PLWH in settings of real clinical practice in Italy to provide a current view of the changes introduced by the advent of B/F/TAF with respect to already used ARTs.

The main finding emerging here was that people who received B/F/TAF showed a general better drug utilization, as they were less likely to interrupt or switch treatment and to be adherent compared to those receiving other ARTs. When this study was set up, most of the available data on B/F/TAF arrived from clinical research. The BRAAVE study, a phase 3b, multicenter, open-label US trial, showed that switching to B/F/TAF was effective in maintaining HIV viral suppression for 48 weeks [28]. A successive investigation, with an observation period extended to 72 weeks, confirmed the positive virologic outcomes reported in the BRAAVE study and also highlighted the benefit of B/F/TAF on adherence, since 74% of the participants had high (PDC ≥ 95%) adherence, 21% had intermediate (PDC ≥ 80 and <95%) adherence, and only 3% had poor (<80%) adherence [29].

In this analysis, the lower uptake of B/F/TAF compared with other ART regimens likely reflects real-world diffusion patterns rather than limited clinical value. B/F/TAF became reimbursable in Italy during the study period, when prescribing practices were still influenced by established regimens, local formularies, and eligibility criteria related to resistance profiles. In this context, its preferential use in a smaller and clinically selected population is consistent with a gradual adoption process. Notably, despite its lower utilization, B/F/TAF was associated with improved adherence, persistence, and reduced non-HIV healthcare resource use, indicating that broader implementation may yield further benefits. From an overview of PLWH included in this analysis, those who received B/F/TAF were a younger population, with a markedly better clinical profile, as documented by the lower CCI and the lower rate of diseases commonly found in concomitance with HIV infection [25,26]. Hence, it might also be postulated that these more favorable conditions might have facilitated adherence to therapy. Evidence has suggested that more complex multi-pill regimens represent one main barrier to adherence, and for this reason the spectrum of fixed-dose combination therapies for HIV is continuously broadening. In this context, the advent of B/F/TAF as STR appears useful to address several clinical management issues, including viral suppression, pretreatment CD4 cell count, HIV drug resistance, DDIs, and adherence-friendliness [30].

B/F/TAF-treated people consistently demonstrated a risk of discontinuing or switching therapy lowered by 34%. Up to now, evidence of persistence on therapy with B/F/TAF from real-life settings is scarce. A single-center retrospective study in Italy on PLWH suggested that therapy with B/F/TAF as STR successfully improved CD4 absolute and percentage cell count, CD4/CD8 ratio, HIV-RNA undetectability and stay-on-therapy [31]. Nevertheless, the improved adherence and persistence observed with B/F/TAF are likely multifactorial and may reflect not only regimen simplicity, but also favorable tolerability, a low potential for drug–drug interactions, and sustained treatment continuity in routine clinical practice. In addition, it was noted that patients treated with B/F/TAF had a more favorable baseline clinical profile, which may have partially contributed to the observed differences despite adjustment through PSM.

Patients treated with B/F/TAF had lower consumption and expenses related to non-HIV-related medications compared to those on other ARTs. In detail, the cost savings observed with B/F/TAF were largely driven by reduced use of non-HIV medications, while differences in hospitalization and outpatient service costs were more limited. These findings are consistent with a recent cost analysis in the US describing significant cost savings in PLWH treated with INI-based STRs, further corroborating the view that optimizing treatment regimens may be a helpful strategy to improve adherence and persistence to treatment and maintain lower healthcare costs [32].

### Limitations of the Study

These results should be interpreted considering some limitations related to the observational retrospective nature of the analysis, which was based on data extrapolated from administrative databases. Since these databases are conceived to track healthcare services and drugs reimbursed by the NHS, there might be lacking or incomplete clinical information on a number of other potential confounders (i.e., disease severity, comorbidities) and this might have weakened the robustness of some results. Additionally, some data like viral load, lymphocyte subsets, CD4 count and CD4/CD8 ratio are not traceable in the databases. Another limitation lies in the imbalance of cohort sizes, reflecting the recent reimbursement of B/F/TAF in Italy and real-world prescribing patterns. To mitigate this issue, PSM was applied, resulting in balanced cohorts for comparative analyses. However, the smaller B/F/TAF sample may limit generalizability, and further studies with larger populations are needed. Another point that deserves attention is the exclusion of patients with gaps between ART prescriptions longer than 12 months, which may have resulted in a study population with more regular treatment patterns, potentially limiting the representativeness of individuals with intermittent care or poor engagement in treatment. These findings should therefore be interpreted with caution, as unmeasured confounders, selection mechanisms related to treatment initiation, the lack of information on reasons for treatment switching (preventing differentiation between simplification-driven switches and those related to tolerability or adverse events), and the absence of certain clinical variables (e.g., virological and immunological markers) may have partially contributed to the observed difference. Moreover, the comparator “other ART” group was heterogeneous and could not be further stratified by regimen composition (e.g., single-tablet versus multi-tablet regimens or by third-agent class), which may have influenced treatment duration and related outcomes. Lastly, the results have been generated from the data of a sample of Italian health-assisted individuals, and this may limit their generalizability on a larger national and international scale.

## 5. Conclusions

This real-world analysis suggests that PLWH who received B/F/TAF were less likely to interrupt or switch treatment and were more frequently adherent compared to those receiving other ARTs. The utilization of B/F/TAF was associated with cost savings for non-HIV-related drugs and hospitalizations. These results provide some new insights for improving the management of PLWH with potential advantages from both people and health systems’ perspectives. Early ART initiation with B/F/TAF in newly diagnosed people with HIV might represent a valuable strategy to reduce future direct and indirect costs of care.

## Figures and Tables

**Table 1:** The main demographic and clinical characteristics of PLWH started on B/F/TAF treatment (naïve B/F/TAF group) and 5576 started on other ARTs (naïve other ART group).

	Naïve B/F/TAF	Naïve Other ART
**N.** **of patients**	374	5576
Age (years), mean ± SD	46.8 ± 11.8	59.5 ± 15.7
Male, n (%)	291 (77.8%)	3490 (62.6%)
CCI, mean ± SD	0.2 ± 0.6	1.2 ± 1.7
—0, n (%)	309 (82.6%)	2475 (44.4%)
—1, n (%)	53 (14.2%)	1357 (24.3%)
—≥2, n (%)	12 (3.2%)	1744 (31.3%)
Depression, n (%)	27 (7.2%)	486 (8.7%)
Respiratory disease, n (%)	50 (13.4%)	1178 (21.1%)
Renal failure, n (%)	N.I.	211 (3.8%)
Alcohol/drugs abuse, n (%)	9 (2.4%)	28 (0.5%)
Osteoporosis, n (%)	N.I.	212 (3.8%)
Cardiovascular disease, n (%)	N.I.	403 (7.2%)
Diabetes, n (%)	20 (5.3%)	841 (15.1%)
Dyslipidemia, n (%)	21 (5.6%)	974 (17.5%)
HBV/HCV, n (%)	15 (4.0%)	960 (17.2%)
Hypertension, n (%)	50 (13.4%)	2363 (42.4%)
Cancer, n (%)	16 (4.3%)	2268 (40.7%)
Follow-up in years, mean ± SD	2.3 ± 0.7	2.5 ± 0.8
Characterization period in years, mean ± SD	6.6 ± 2.7	6.6 ± 2.9

Abbreviations: ART, antiretroviral therapy; B/F/TAF, bictegravir/emtricitabine/tenofovir; CCI, Charlson comorbidity index; HBV, hepatitis B virus; HCV, hepatitis C virus; SD, standard deviation.

**Table 2:** Cox regression model for predictors of therapy interruptions/switches in PLWH. Significant *p* values are highlighted in bold.

	HR	95% CI		*p*
B/F/TAF (Ref.: other ART)	0.663	0.574	0.765	**<0.001**
Age class (Ref.: 18–44 years)				
45–59 years	0.713	0.650	0.782	**<0.001**
60–79 years	0.618	0.556	0.687	**<0.001**
≥80 years	0.635	0.537	0.751	**<0.001**
Male sex	0.954	0.887	1.025	0.195
CCI (Ref.: 0)				
1	0.930	0.835	1.036	0.186
≥2	0.954	0.853	1.066	0.405
Depression	1.037	0.914	1.177	0.572
Respiratory disease	0.967	0.879	1.064	0.491
Renal failure	1.403	1.157	1.701	**0.001**
Alcohol/drugs abuse	1.142	0.769	1.695	0.511
Osteoporosis	0.883	0.725	1.074	0.212
Cardiovascular disease	0.914	0.777	1.077	0.283
Diabetes	1.076	0.961	1.204	0.205
Dyslipidemia	0.910	0.814	1.017	0.095
HBV/HCV	0.672	0.597	0.756	**<0.001**
Hypertension	0.964	0.883	1.053	0.419
Cancer	0.889	0.809	0.977	**0.015**

Abbreviations: ART, antiretroviral therapy; B/F/TAF, bictegravir/emtricitabine/tenofovir; CCI, Charlson comorbidity index; CI, confidence interval; HR, hazard ratio; HBV, hepatitis B virus; HCV, hepatitis C virus.

**Table 3:** Logistic regression model for predictors of therapy interruptions in PLWH. Significant *p* values are highlighted in bold.

	HR	95% CI		*p*
B/F/TAF (Ref.: other ART)	**0.708**	**0.974**	**−2.280**	**0.023**
Age class (Ref.: 18–44 years)				
45–59 years	**0.682**	**0.850**	**−4.870**	**<0.001**
60–79 years	**0.643**	**0.821**	**−5.120**	**<0.001**
≥80 years	**0.669**	**0.963**	**−2.360**	**0.018**
Male sex	**0.841**	**0.988**	**−2.260**	**0.024**
CCI (Ref.: 0)				
1	0.903	1.149	0.300	0.763
≥2	0.931	1.186	0.800	0.422
Depression	0.832	1.110	−0.540	0.591
Respiratory disease	0.829	1.022	−1.550	0.120
Renal failure	**1.129**	**1.718**	**3.100**	**0.002**
Alcohol/drugs abuse	0.727	1.845	0.620	0.537
Osteoporosis	0.745	1.130	−0.810	0.416
Cardiovascular disease	0.784	1.114	−0.750	0.452
Diabetes	0.934	1.193	0.860	0.390
Dyslipidemia	0.816	1.038	−1.350	0.178
HBV/HCV	**0.714**	**0.918**	**−3.290**	**0.001**
Hypertension	0.890	1.082	−0.380	0.705
Cancer	0.952	1.170	1.030	0.303

Abbreviations: ART, antiretroviral therapy; B/F/TAF, bictegravir/emtricitabine/tenofovir; CCI, Charlson comorbidity index; CI, confidence interval; HBV, hepatitis B virus; HCV, hepatitis C virus; HR, hazard ratio.

**Table 4:** Cox regression model for predictors of switch in PLWH. Significant *p* values are highlighted in bold.

	HR	95% CI		*p*
B/F/TAF (Ref.: other ART)	**0.301**	**0.221**	**0.409**	**<0.001**
Age class (Ref.: 18–44 years)				
45–59 years	**0.680**	**0.583**	**0.792**	**<0.001**
60–79 years	**0.400**	**0.326**	**0.490**	**<0.001**
≥80 years	**0.213**	**0.134**	**0.340**	**<0.001**
Male sex	1.120	0.973	1.291	0.115
CCI (Ref.: 0)				
1	**0.756**	**0.607**	**0.941**	**0.012**
≥2	**0.581**	**0.443**	**0.762**	**<0.001**
Depression	1.181	0.927	1.506	0.179
Respiratory disease	1.167	0.955	1.427	0.132
Renal failure	**1.717**	**1.137**	**2.592**	**0.010**
Alcohol/drugs abuse	1.005	0.500	2.021	0.989
Osteoporosis	0.786	0.487	1.267	0.323
Cardiovascular disease	0.975	0.677	1.404	0.891
Diabetes	1.065	0.822	1.381	0.632
Dyslipidemia	0.954	0.748	1.217	0.704
HBV/HCV	**0.307**	**0.230**	**0.411**	**<0.001**
Hypertension	0.876	0.727	1.055	0.163
Cancer	**0.423**	**0.337**	**0.529**	**<0.001**

Abbreviations: ART, antiretroviral therapy; B/F/TAF, bictegravir/emtricitabine/tenofovir; CCI, Charlson comorbidity index; CI, confidence interval; HBV, hepatitis B virus; HCV, hepatitis C virus; HR, hazard ratio.

**Table 5:** Cox regression models for predictors of adherence in PLWH, with PDC ≥ 85% and PDC ≥ 95% as cut-offs for adherence. Significant *p* values are highlighted in bold.

	Adherence, PDC ≥ 85%				Adherence, PDC ≥ 95%			
	HR	95% CI		*p*	HR	95% CI		*p*
B/F/TAF (Ref.: other ART)	**2.401**	**1.584**	**3.641**	**<0.001**	**1.776**	**1.278**	**2.469**	**0.001**
Age class (Ref.: 18–44 years)								
45–59 years	**1.386**	**1.078**	**1.783**	**0.011**	1.197	0.959	1.496	0.112
60–79 years	**1.481**	**1.124**	**1.952**	**0.005**	1.223	0.960	1.560	0.104
≥80 years	1.193	0.804	1.772	0.380	1.065	0.750	1.512	0.723
Male sex	0.967	0.809	1.156	0.711	0.986	0.845	1.151	0.858
CCI (Ref.: 0)								
1	0.802	0.619	1.039	0.095	0.886	0.706	1.112	0.297
≥2	0.882	0.678	1.148	0.352	**0.774**	**0.618**	**0.970**	**0.026**
Depression	0.977	0.718	1.328	0.880	0.933	0.717	1.215	0.607
Respiratory disease	0.857	0.687	1.070	0.173	0.897	0.740	1.088	0.271
Renal failure	**0.386**	**0.251**	**0.592**	**<0.001**	**0.424**	**0.282**	**0.636**	**<0.001**
Alcohol/drugs abuse	1.164	0.331	4.097	0.813	1.896	0.541	6.643	0.317
Osteoporosis	1.191	0.761	1.862	0.445	1.188	0.810	1.743	0.377
Cardiovascular disease	1.205	0.825	1.761	0.335	1.227	0.884	1.703	0.221
Diabetes	0.969	0.743	1.264	0.816	1.057	0.838	1.333	0.641
Dyslipidemia	1.108	0.856	1.435	0.436	1.064	0.852	1.328	0.586
HBV/HCV	**2.296**	**1.719**	**3.068**	**<0.001**	**2.037**	**1.596**	**2.601**	**<0.001**
Hypertension	0.922	0.747	1.138	0.448	0.960	0.801	1.151	0.662
Cancer	1.209	0.967	1.512	0.096	1.110	0.915	1.347	0.291

Abbreviations: ART, antiretroviral therapy; B/F/TAF, bictegravir/emtricitabine/tenofovir; CCI, Charlson comorbidity index; CI, confidence interval; HBV, hepatitis B virus; HCV, hepatitis C virus; HR, hazard ratio; PDC, proportion of days covered.

**Table 6:** Main demographic and clinical characteristics of PSM-balanced B/F/TAF PLWH, other ART-treated PLWH and non-HIV-controls. SMD values > 2 are highlighted in bold.

	Non-HIV-Controls	B/F/TAF Group	Other ART Group	SMD
**N** **of patients**	**321**	**321**	**321**	
Index year, n (%)				0.17
—2019	20 (6.2%)	17 (5.3%)	32 (10.0%)	
—2020	179 (55.8%)	158 (49.2%)	161 (50.2%)	
—2021	122 (38.0%)	146 (45.5%)	128 (39.9%)	
Age (years), mean ± SD	46.1 ± 15.3	46.5 ± 11.8	47.0 ± 14.6	0.14
Male, n (%)	187 (58.3%)	254 (79.1%)	213 (66.4%)	**0.31**
CCI, mean ± SD	0.1 ± 0.3	0.2 ± 0.6	0.1 ± 0.4	0.17
—0, n (%)	286 (89.1%)	267 (83.2%)	285 (88.8%)	
—1, n (%)	34 (10.6%)	45 (14.0%)	33 (10.3%)	
—≥2, n (%)	NI	9 (2.8%)	NI	
Depression, n (%)	13 (4.0%)	23 (7.2%)	10 (3.1%)	0.12
Respiratory disease, n (%)	28 (8.7%)	41 (12.8%)	27 (8.4%)	0.09
Renal failure, n (%)	0 (0.0%)	NI	0 (0.0%)	0.05
Alcohol/drugs abuse, n (%)	NI	6 (1.9%)	NI	0.10
Osteoporosis, n (%)	NI	NI	NI	0.00
Cardiovascular disease, n (%)	NI	NI	6 (1.9%)	0.05
Diabetes, n (%)	15 (4.7%)	16 (5.0%)	11 (3.4%)	0.05
Dyslipidemia, n (%)	25 (7.8%)	18 (5.6%)	22 (6.9%)	0.06
HBV/HCV, n (%)	NI	13 (4.0%)	NI	0.13
Hypertension, n (%)	47 (14.6%)	41 (12.8%)	51 (15.9%)	0.06
Cancer, n (%)	13 (4.0%)	12 (3.7%)	14 (4.4%)	0.02

Abbreviations: ART, antiretroviral therapy; B/F/TAF, bictegravir/emtricitabine/tenofovir; CCI, Charlson comorbidity index; HBV, hepatitis B virus; HCV, hepatitis C virus; HIV, human immunodeficiency virus; SD, standard deviation; SMD, standardized mean difference.

**Table 7:** Healthcare resources consumption in PSM-matched cohorts. Significant *p* values are highlighted in bold.

N. of Prescriptions/Provisions per Person, Mean ± SD	B/F/TAF Cohort	Other ART Cohort	*p* Value
**N. of patients**	**321**	**321**	
Prescriptions, non-HIV-related	6.0 ± 8.4	8.8 ± 12.2	**<0.001**
Hospitalizations, non-HIV-related	0.2 ± 0.5	0.3 ± 0.8	**0.050**
Outpatient services	7.8 ± 6.4	8.6 ± 10.4	0.228

Abbreviations: ART, antiretroviral therapy; B/F/TAF, bictegravir/emtricitabine/tenofovir; HIV, human immunodeficiency virus; SD, standard deviation.

**Table 8:** Healthcare direct costs (outliers excluded) in PSM-matched cohorts during the first 12 months of follow-up.

Cost (€) per Person	B/F/TAF Cohort	Other ART Cohort	*p* Value
**N. of patients**	**319**	**307**	
Prescriptions, non-HIV-related, mean ± SD	572 ± 1853	1553 ± 4225	**<0.001**
Hospitalizations, non-HIV-related, mean ± SD	445 ± 1960	710 ± 2358	0.125
Outpatient services, mean ± SD	1061 ± 1319	938 ± 1306	0.242
Total, mean ± SD	2078 ± 3292	3202 ± 5850	**0.003**
Prescriptions, non-HIV-related, median (IQR)	56.0 (236.0)	42.0 (454.0)	0.624
Hospitalizations, non-HIV-related, median (IQR)	0.0 (0.0)	0.0 (0.0)	0.393
Outpatient services, median (IQR)	754.0 (1031.0)	543.0 (940.0)	**0.003**
Total, mean ± SD median (IQR)	1026.0 (1517.0)	837.0 (2121.0)	0.201

Abbreviations: ART, antiretroviral therapy; B/F/TAF, bictegravir/emtricitabine/tenofovir; HIV, human immunodeficiency virus; IQR, interquartile range; SD, standard deviation.

## Data Availability

The data supporting the findings of this article are available at an aggregated level from the authors upon reasonable request and with permission from the participating healthcare entities. Requests for access should be directed to the corresponding author.

## Abbreviations

The following abbreviations are used in this manuscript:

ART	Antiretroviral Therapies
ATC	Anatomical-Therapeutic Chemical
B/F/TAF	Bictegravir/emtricitabine/tenofovir
CCI	Charlson Comorbidity Index
CI	Confidence Interval
DDIs	Drug–Drug Interactions
DRG	Diagnosis-Related Group
HBV	Hepatitis B Virus
HCV	Hepatitis C Virus
HIV	Human Immunodeficiency Virus
HR	Hazard Ratio
ICD-9-CM	International Classification of Diseases, Ninth Revision, Clinical Modification
INI	Integrase Inhibitor
IQR	Interquartile range
LHUs	Local Health Units
MTRs	Multi-Tablet Regimen
NHS	National Health System
NI	Not Issuable
PDC	Proportion of Days Covered
PLWH	People Living With HIV
PSM	Propensity Score Matching
SD	Standard Deviation
SMD	Standardized Mean Difference
STR	Single-Tablet Regimen
